# NBP Cytoprotective Effects Promoting Neuronal Differentiation in BMSCs by Inhibiting the p65/Hes1 Pathway

**DOI:** 10.5812/ijpr-132496

**Published:** 2023-07-11

**Authors:** Eryi Zhao, Peijian Huang, Zhongyan Zhao, Shixiong Huang, Shijun Hu, Ling Xie, Jie Lin, Daimei Wang

**Affiliations:** 1 Hainan General Hospital, Hainan Medical University, Haikou, China

**Keywords:** 3-N-butylphthalide, Bone Marrow-Derived Mesenchymal Stem Cells, Neuronal Differentiation, p65/HES 1 Pathway

## Abstract

**Background:**

Bone marrow-derived mesenchymal stem cell (BMSC) transplantation has become an effective method for treating neurodegenerative diseases.

**Objectives:**

This study investigated the effect of 3-N-butylphthalide (NBP) on the neuronal differentiation of BMSCs and its potential mechanism.

**Methods:**

In this study, a 3-(4,5-dimethylthiazol-2-yl)-2,5-diphenyltetrazolium bromide assay was performed to detect cell proliferation and terminal deoxynucleotidyl transferase dUTP nick-end labeling (TUNEL) staining was conducted to detect the apoptosis of BMSCs. Quantitative real-time polymerase chain reaction (RT-qPCR) and Western blot analysis were performed to detect the messenger RNA (mRNA) and protein expression levels, respectively. An enzyme-linked immunosorbent serologic assay assessed the levels of interleukin-1β, tumor necrosis factor-α, and cyclic adenosine monophosphate (cAMP). Moreover, a flow cytometry assay was used to detect the proportion of active β-tubulin III (TUJ-1) cells, and TUJ-1 expression was observed by immunofluorescence assay.

**Results:**

The results showed that a low concentration of NBP promoted the proliferation and induction of BMSC neuronal differentiation while inhibiting apoptosis, the production of inflammatory factors, and p65 expression. Compared with differentiation induction alone, combined NBP treatment increased the levels of nestin, neuron-specific enolase (NSE), TUJ-1, and microtubule-associated protein 2 (MAP2) protein, as well as the ratio of TUJ-1-positive cells and cAMP expression. Furthermore, p65 overexpression weakened the effect of NBP, and the overexpression of hairy and enhancer of split homolog-1 (HES1) reversed the effect of NBP in the induction of BMSC neuronal differentiation in vitro.

**Conclusions:**

We confirmed that NBP exhibited potential therapeutic properties in the stem cell transplantation treatment of neurodegenerative diseases by protecting cells and promoting BMSC neuronal differentiation by inhibiting the p65/HES 1 pathway.

## 1. Background

Neurodegenerative diseases, characterized by the progressive loss of neurons, nerve cell damage, and neurologic impairment, are a huge burden to individuals and society. These include Parkinson's disease, amyotrophic lateral sclerosis, Alzheimer's, spinal cord injury, and stroke (1). In recent years, stem cell transplantation has become the focus of increasing attention as a new method for treating nervous system degenerative diseases (2).

Bone marrow-derived mesenchymal stem cells (BMSCs) are pluripotent stem cells from bone marrow and are a good source of transplanted stem cells, potentially differentiating into various related tissues and cells (3). Under different conditions, they can greatly expand in vitro and differentiate into various cell types, such as osteoblasts, adipocytes, and neural cells (4). Researchers have reported that BMSCs can be differentiated into neuron-like cells in vitro under specific induction culture conditions (5-7). Relative to embryonic stem cells and neural stem cells, BMSCs have greater potential as therapeutic agents, as they are easy to isolate and amplify, with strong self-renewal capabilities, pluripotent differentiation ability, and low immunogenicity (4). However, only a few BMSCs can successfully differentiate into neurons in vitro, a limitation of stem cell transplantation treatment in neurodegenerative diseases. Therefore, there is an urgent need for an effective way to improve the neuronal differentiation ability of BMSCs.

Dl-3-N-butylphthalide (NBP), one of the chemical components from the seed of *Apium graveolens* Linn, has been approved and clinically used to treat stroke in China (8). Recently, studies have shown that NBP may exert potential pharmacologic effects and protect neural cells by inhibiting inflammatory responses, oxidative stress, and apoptosis (9-12). Que et al. reported that NBP rescued dopaminergic neurons by inhibiting the NOD-like receptor family pyrin domain containing 3 (NLRP3) inflammasome, ameliorating mitochondrial impairment in a Parkinson's disease model (13). Yang et al. (14) demonstrated that NBP may play a neuroprotective role in cardiac arrest, followed by cardiopulmonary resuscitation. These studies suggested that NBP has a neuroprotective function and potential therapeutic effects on various neurodegenerative diseases. However, whether NBP promotes the neuronal differentiation of transplanted BMSCs remains unclear.

## 2. Objectives

Our study aimed to investigate the role of NBP in the neuronal differentiation of BMSCs in vitro.

## 3. Methods

### 3.1. Isolation, Culture, and Induction of BMSCs

Briefly, rats were euthanized by the controlled inhalation of CO_2_, and the bone marrow was flushed from the hind legs with low-glucose Dulbecco's modified eagle medium (DMEM) with 10% fetal bovine serum (FBS) (15). Then, the BMSCs were collected and cultured on a 10-cm diameter plate until 80% - 90% confluent. After dissociation with 0.25% trypsin, purified fourth-generation rat marrow-derived mesenchymal stem cells (rMSCs) were obtained and added to a 10% FBS DMEM medium for subsequent analysis. The BSMSs were tested by flow cytometry analysis using a cluster of differentiation - 29 (CD29), CD44, and CD45, and the results are shown in Appendix 1 of the Supplementary File. The induction of BMSCs to neural cells was performed with 2 days of pre-induction by a DMEM medium with 1-mM b-mercaptoethanol, followed by 6 days of induction by DMEM medium with 2% the human leukocyte antigen class I molecule (B27), 10-ng/mL fibroblast growth factor 2 (FGF2) and 10-ng/mL epidermal growth factor (EGF).

### 3.2. Cell Transfection

The experiments were performed as previously described (16). The full-length sequence of the p65 gene (NC_051336.1, in the NCBI) and Hes1 gene (NC_051346.1, in the NCBI) was synthesized and subcloned into pcDNA 3.1 vector (GenePharma, Shanghai, P.R. China). Bone marrow-derived mesenchymal stem cells cells were seeded into six-well plates the day prior to transfection. When the cell reached 80% confluence, the pcDNA 3.1-p65, pcDNA 3.1-Hes1, and Hes1 shRNA plasmid (GenePharma, Shanghai, China) were transfected as 1:100 into BMSCs cells to obtain stable p65 or Hes1 overexpression and Hes1 knockdown cells by using Lipofectamine 2000 (Invitrogen, USA). Transfected cells were collected for subsequent study 48 h after transfection.

Cell viability was determined using trypan blue staining. When the cell confluence reached 80%, the BMSCs were transfected with a plasmid (GenePharma, Shanghai, China) using Lipofectamine 2000 (Invitrogen, Life Technologies, Carlsbad, CA, USA) according to the manufacturer's instructions in a six-well plate. To obtain stable p65-overexpression cells, the cells were transfected with the pcDNA 3.1-p65 plasmid and incubated in a growth medium containing 200-μg/mL hygromycin B (Invitrogen) at room temperature for 20 min. To obtain stable Hes1-overexpression cells, the cells were transfected with the pcDNA 3.1-Hes1 plasmid and incubated in a growth medium containing 200-μg/mL hygromycin B (Invitrogen) at room temperature for 20 min. To obtain stable Hes1-knockdown cells, the cells were transfected with the Hes1 shRNA (targeting sequences: GTGAAAGTCTCAAGTAAAAGAGA) plasmid and incubated in a growth medium containing 200-μg/mL hygromycin B (Invitrogen) at room temperature for 20 min.

### 3.3. MTT Assay

Cell viability was analyzed by 3-(4,5-dimethylthiazol-2-yl)-2,5-diphenyltetrazolium bromide (MTT) assay according to the instructions (17). BMSCs were seeded into 96-well plates at a cell density of 1 × 10^5^ cells/well and cultured in DMEM with 10% FBS at 37°C for 24, 48, and 72 h, respectively. Then, 50-μL MTT (5 mg/mL, Sigma-Aldrich, St. Louis, MO) was pipetted into each well. After 4 h of incubation, 200-μL Dimethyl sulfoxide (DMSO) was added to terminate the reaction. The absorbance was detected by a fluorescent microplate reader at a wavelength of 490 nm.

### 3.4. Enzyme-Linked-Immunosorbent Serologic Assay

Twenty-four hours after NBP treatment, the cell culture medium supernatant was collected and centrifuged. A protein extraction reagent was used to lyse the BMSCs; then, rat interleukin-1β (IL-1β) and tumor necrosis factor-α (TNF-α) enzyme-linked-immunosorbent serologic assay (ELISAs) kits (MLbio, Shanghai, China) were employed to detect the concentration of inflammatory factors. A cyclic adenosine monophosphate (cAMP) ELISA kit (MLbio) was used to evaluate the cAMP level (as a neuron function molecule) according to the instructions.

### 3.5. Western Blot Assay

Experiments were performed according to the manufacturer’s instructions and standard procedures. The total proteins of the BMSCs were isolated by a cell lysis buffer (Beyotime, Nanjing, China), and the total protein concentrations were quantified using a bicinchoninic (BCA) protein assay reagent kit (Bio-Rad, Hercules, CA, USA). Next, 20 μg of extracted protein was loaded onto polyacrylamide -sodium dodecyl sulfate (SDS) gels and transferred onto a polyvinylidene fluoride membrane. The membranes were blocked with 5% non-fat milk. They were incubated with a primary antibody against caspase 3 (Abcam, Cambridge, UK, ab184787), B-cell lymphoma 2 (Bcl-2) (Abcam, ab194583), Bcl-2-associated X (Bax) (Abcam, ab182734), p65 (Abcam, ab194726), Hes1 (Abcam, ab108937), nestin (Abcam), neuron-specific enolase (NSE) (Sigma-Aldrich, SAB4200347), TUJ-1 (Abcam, ab18207) and microtubule-associated protein 2 (MAP2) (Sigma-Aldrich, ZRB2290) overnight at 4°C. Subsequently, the membranes were incubated with secondary antibody goat anti-rabbit IgG H & L (Abcam, ab6721) for 2 h at 4°C. The intensity of protein expression was detected by electrogenerated chemiluminescence (ECL) and quantified by Image J Software (ImageJ Software Inc., USA).

### 3.6. Quantitative Real-time Polymerase Chain Reaction Assay

The experiments were performed according to the manufacturer’s instructions and standard procedures. The reagent TRIzol (Invitrogen, Thermo Fisher Scientific, USA) was used to extract the total RNA. Then, the RNA was reverse transcribed into cDNA using a commercial kit (One-step RT-PCR Kit, Biolab, Beijing, China). For the real-time polymerase chain reaction (RT-PCR), SYBR Green PCR Master Mix (Takara, Kusatsu, Japan) was used. The housekeeping gene glyceraldehyde-3-phosphate dehydrogenase was used as an internal reference to measure the expression of the target gene. The experiment was repeated three times, and 2^-ΔΔCt^ methods were used to calculate the relative expression. The primer sequences are presented in Appendix 3 of the Supplementary materials.

### 3.7. Flow Cytometry Analysis

Flow cytometry was utilized to detect the proportion of TUJ-1-positive cells. The cells were digested with 0.25% trypsin, collected, and re-suspended in PBS containing 0.1% Triton X-100 in 1-mL 4% paraformaldehyde solution for 3 min. Next, the cells were sealed for 30 min at 37°C with sheep serum and re-suspended with primary antibody against TUJ-1 (Abcam, ab18207). Subsequently, the cells were incubated with goat anti-rabbit IgG H&L (FITC) (Abcam, ab6717 secondary antibody) for 30 min at 4°C. The cells were analyzed using flow cytometry (Leica, Wetzlar, Germany). Each experiment was performed in triplicate. In addition, apoptosis was also analyzed via flow cytometry. The process was conducted according to the instructions of an Annexin V-FITC·PI Double-Stain Apoptosis Detection (Invitrogen).

### 3.8. Statistical Analysis

All the investigations were performed independently at least three times. Data were expressed as means ± standard deviation of the mean (SD) and analyzed by SAPS 18.0 software. Statistical significance was determined using a two-tailed unpaired Student's *t*-test and a one- or two-way analysis of variance (ANOVA). The Student's *t*-test compares the means between two groups, whereas ANOVA is used to compare the means among three or more groups. A value of P < 0.05 was considered statistically significant.

## 4. Results

### 4.1. NBP Influencing the Proliferation and Apoptosis of BMSCs

**Figure 1.**
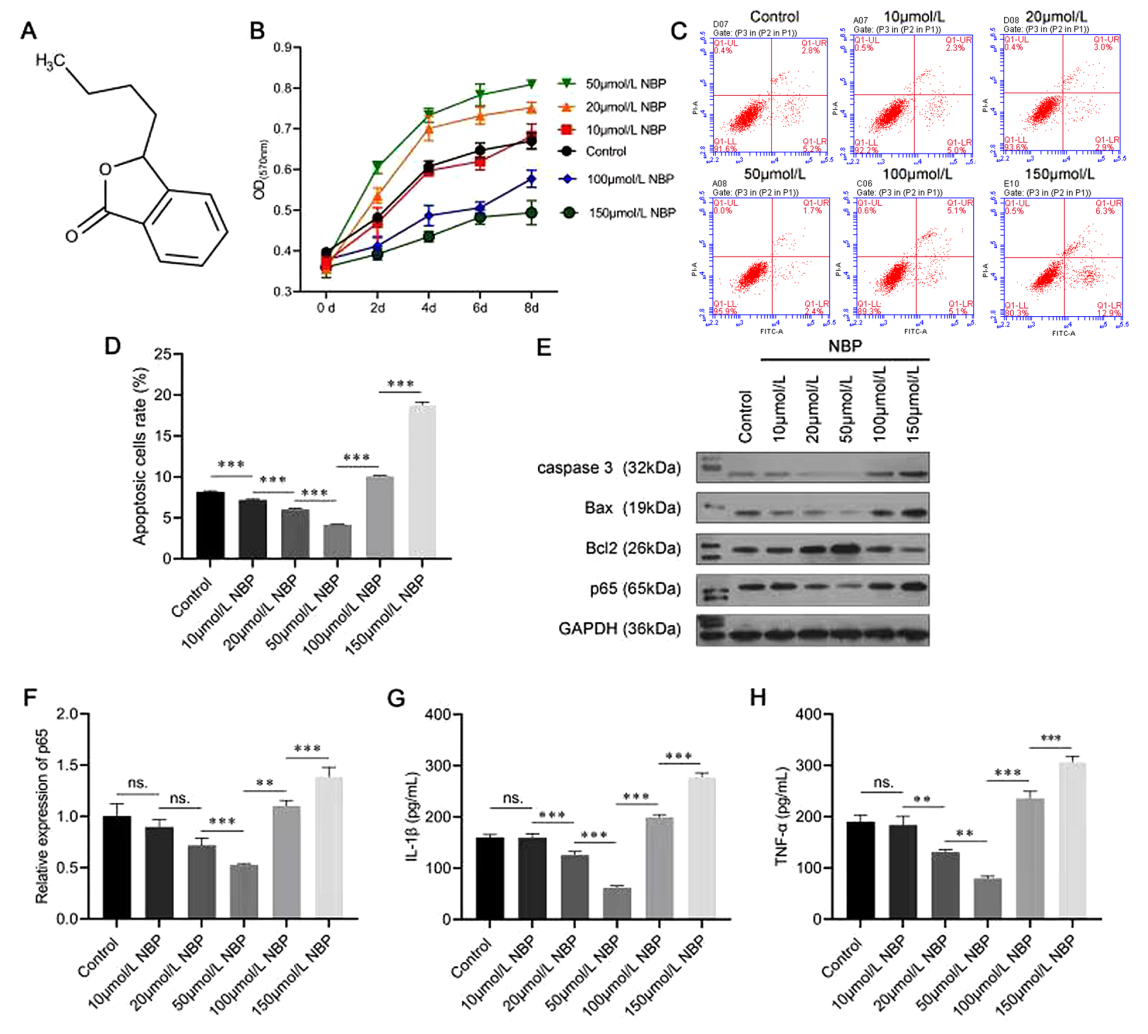
Effects of 3-N-butylphthalide (NBP) on the proliferation and apoptosis of bone marrow-derived mesenchymal stem cells (BMSCs). A, the molecular structure of NBP; B, MTT assay was performed to assess the viability of BMSCs; C, D, flow cytometry was used to evaluate apoptosis; E, Western blot assay evaluated caspase-3, Bcl-2-associated X (Bax), B-cell lymphoma 2 (Bcl-2), and p65 protein levels; F, the messenger RNA (mRNA) expression of p65 was evaluated using an real-time polymerase chain reaction (RT-PCR) assay; G, H, the enzyme-linked-immunosorbent serologic assay (ELISA) was carried out to evaluate the expressions of interleukin-1β (IL-1β) and tumor necrosis factor-α (TNF-α) in BMSCs. ns, not significant. ** P < 0.01, *** P < 0.001.

The molecular structure of NBP is shown in Figure 1A. A series of experiments were performed to evaluate whether NBP affected the proliferation and apoptosis of BMSCs. The data from the MTT assay indicated that a low concentration of NBP notably increased the viability of the BMSCs in a dose-dependent manner (20 and 50 µmol/L). In comparison, a high concentration of NBP (100 and 150 µmol/L) significantly inhibited their viability (Figure 1B). As shown in Figure 1C and D, the ratio of apoptotic BMSCs cells was decreased under low-concentration NBP treatment (10, 20, and 50 µmol/L) and increased under high-concentration NBP treatment (100 and 150 µmol/L).

In addition, Western blot analysis was performed to evaluate the effects of NBP on apoptosis-related proteins. The results corresponded with the flow cytometry analysis in that 20- and 50-μmol/L NBP inhibited the protein levels of caspase-3 and Bax and promoted the expression level of Bcl-2; however, 100- and 150-μmol/L NBP had the opposite effect (Figure 1E). The Western blot analysis and the RT-PCR experiment results also showed that the expression of p65 was inhibited at low concentrations and promoted at high concentrations of NBP (Figure 1E and F). Moreover, the ELISA results indicated that a low concentration of NBP (20 and 50 µmol/L) reduced the secretion of both IL-1β and TNF-α, while high-concentration (100 and 150 µmol/L) NBP treatment had opposite effects (Figure 1G and H). These results confirmed that a low concentration of NBP played a protective role for BMSCs by promoting proliferation and inhibiting apoptosis, p65 expression, and inflammatory factor secretion. In contrast, a high concentration of NBP might have toxic effects. As a result, a concentration of 50-µmol/L NBP was selected for the subsequent experiments.

### 4.2. NBP Promoting BMSC Neuronal Differentiation in Vitro

**Figure 2.**
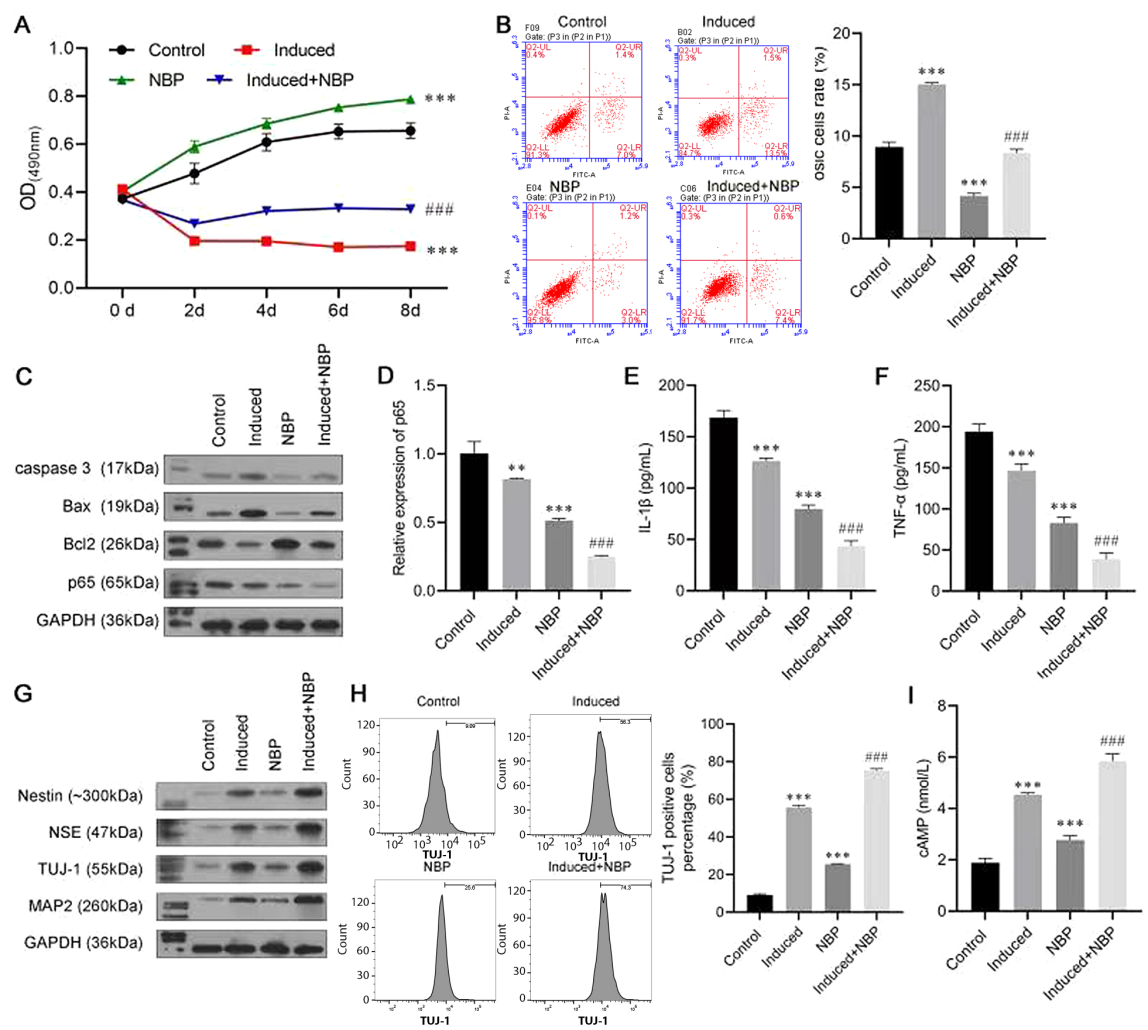
3-N-butylphthalide (NBP) combined treatment promoting bone marrow-derived mesenchymal stem cells (BMSCs) neural differentiation. A, MTT assay was used to investigate the role of NBP in the viability of BMSCs; B, flow cytometry was used to detect apoptosis of BMSCs; C, Western blot assay evaluated the protein levels of caspase-3, Bcl-2-associated X (Bax), B-cell lymphoma 2 (Bcl-2), and p65; D, the messenger RNA (mRNA) expression of p65 was evaluated by real-time polymerase chain reaction (RT-PCR) assay; E, F, enzyme-linked-immunosorbent serologic assay was conducted to detect the levels of interleukin-1β (IL-1β) and tumor necrosis factor-α (TNF-α); G, Western blot assay was carried out to evaluate the expressions of Nestin, neuron-specific enolase (NSE), β-tubulin III (TUJ-1), and microtubule-associated protein 2 (MAP2); H, the proportion of TUJ-1-positive cells was detected by flow cytometry; I, enzyme-linked-immunosorbent serologic assay (ELISA) was conducted to detect the levels of cyclic adenosine monophosphate (cAMP). ** P < 0.01, *** P < 0.001 vs. control group. ## P < 0.01, ### P < 0.001 vs. induce group.

To investigate the role of NBP in neuronal differentiation, BMSCs were induced to differentiate, with or without NBP. The results of the MTT assay are shown in Figure 2A. In the first two days, the cell viability of the Induced group and the Induced + NBP group decreased, which may have been caused by the cytotoxicity of b-mercaptoethanol. However, simulation with NBP increased cell activity, which indicated the protective effect of NBP. Meanwhile, the NBP-alone group had fewer apoptosis cells than the Control group, and the apoptosis rate in the induced + NBP group was significantly lower than that of the Induced group (Figure 2B). The Western blot assay results consistently revealed that NBP could decrease the protein levels of caspase-3 and Bax and increase the level of Bcl-2 expression (Figure 2C). Moreover, p65 expression was inhibited mostly in the Induced + NBP group at protein and RNA levels (Figure 2C and D). The ELISA results showed that the level of IL-1β and TNF-α in the Induced + NBP group were notably lower than those of the NBP and Induced groups (Figure 2E and F).

A Western blot assay was conducted to evaluate the expressions of nerve cell biomarkers, including nestin, NSE, TUJ-1, and MAP2. The induction treatment increased the levels of nestin, NSE, TUJ-1, and MAP2 protein expression compared with those of the control group, while the expression level of these proteins was further promoted in the induced + NBP group, meaning that NBP enhanced the effect of inducing differentiation to neurons (Figure 2G). In addition, the result of flow cytometry also confirmed our findings, in which TUJ-1-positive cells in the induced + NBP group were more abundant than in the Induced and NBP groups (Figure 2H). Furthermore, as a neuron function biomarker, the expression of cAMP in the induced + NBP group was higher than that in the Induced and NBP groups (Figure 2I).

### 4.3. Overexpression of p65 Attenuating the Effect of NBP on BMSC Neuronal Differentiation

**Figure 3.**
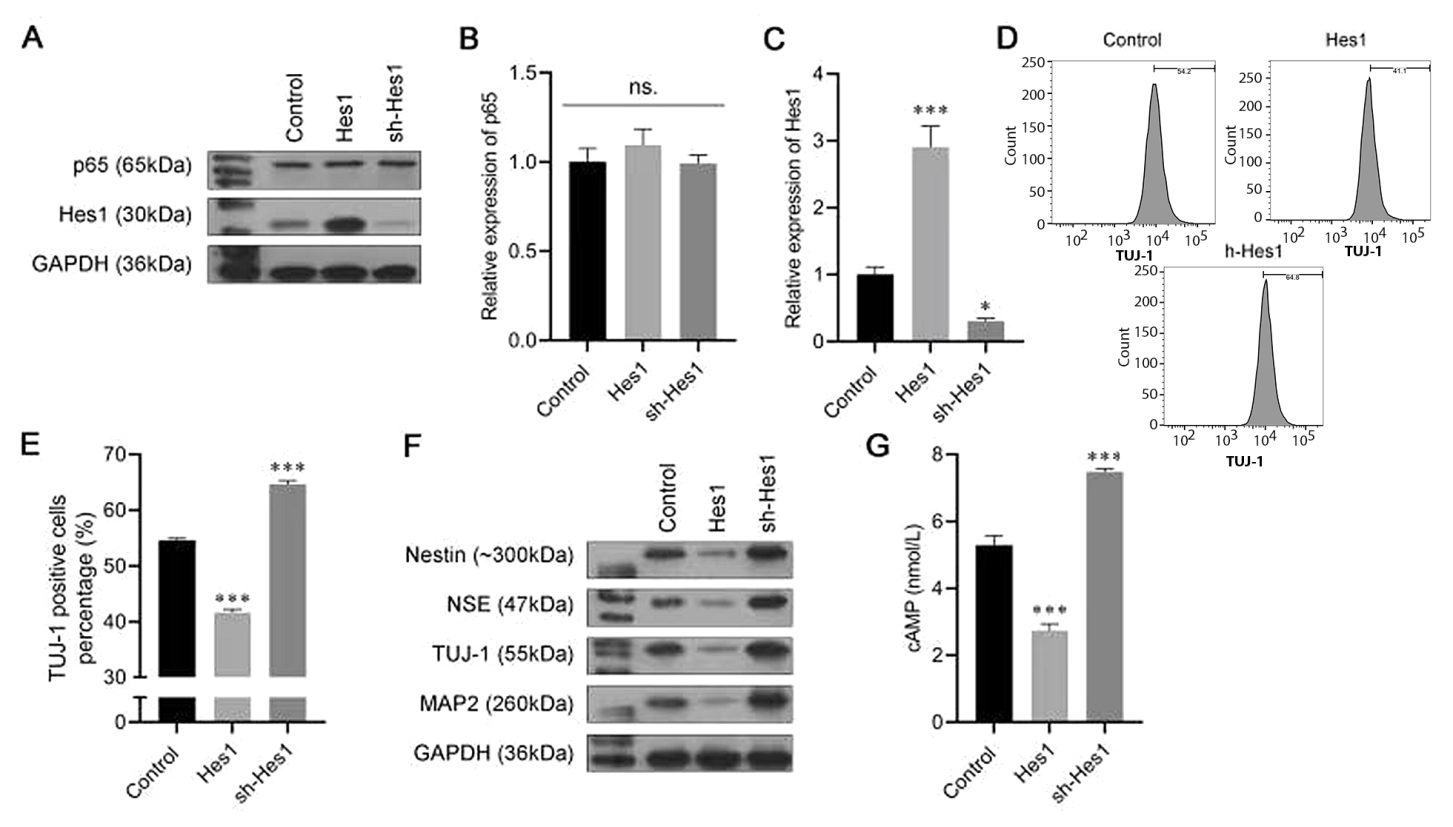
3-N-butylphthalide (NBP) promotes bone marrow-derived mesenchymal stem cells (BMSCs) neural differentiation by inhibiting p65. A, the protein expression of p65 and Hes1 were analyzed using a western blot assay; B, C, the messenger RNA (mRNA) expression of p65 and Hes1 were analyzed using an real-time polymerase chain reaction (RT-PCR) assay; D, E, the proportion of β-tubulin III (TUJ-1)-positive cells was detected by flow cytometry; F, Western blot assay was carried out to evaluate the expressions of Nestin, neuron-specific enolase (NSE), TUJ-1, and microtubule-associated protein 2 (MAP2); G, cyclic adenosine monophosphate (cAMP) expression was assessed by enzyme-linked-immunosorbent serologic assay (ELISA). ns, not significant. * P < 0.05, *** P < 0.001 vs. control group.

To further explore the specific mechanisms of NBP in the progression of neuronal differentiation, we constructed the p65 overexpression plasmid. Transfected with the p65 overexpression plasmid or vector, bone marrow-derived mesenchymal stem cells were induced to differentiate, with or without NBP treatment. Western blot and RT-PCR analyses revealed that after single transfection with the p65 plasmid, the mRNA and protein expression of p65 increased, indicating the successful overexpression of p65 (Figure 3A and B). Meanwhile, we found that the expression of Hes1 was also decreased by NBP and increased by p65 overexpression (Figure 3A and C). The flow cytometry assay revealed that the TUJ-1-positive cell ratio was increased by NBP, while it was recovered by p65 overexpression (Figure 3D and E). As for the neuronal differentiation biomarkers, the protein levels of nestin, NSE, TUJ-1, and MAP2 enhanced by NBP were also reversed by p65 overexpression (Figure 3F). Finally, the ELISA results demonstrated that NBP induced the expression of cAMP, while the effect was reversed by transfection with the p65 overexpression plasmid (Figure 3G). These results demonstrated, first, that NBP induces neuronal differentiation in BMSCs by inhibiting p65. Second, Hes1 might also play a role in this process.

### 4.4. Effect of Hes1 in the Progression of BMSC Neuronal Differentiation

**Figure 4.**
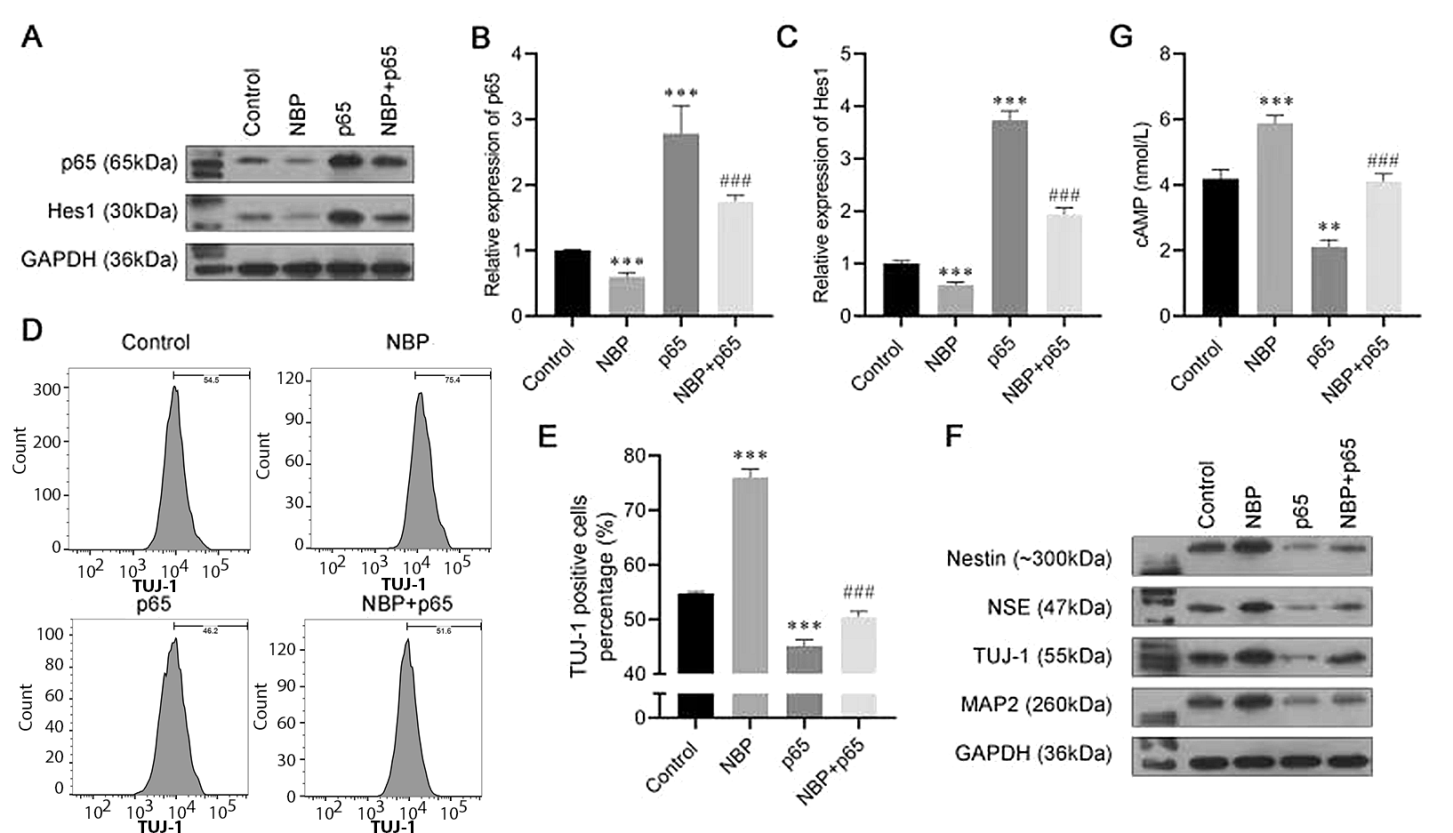
Effect of Hes1 in the progression of bone marrow-derived mesenchymal stem cells (BMSCs) neural differentiation. A, the protein expression of p65 and Hes1 were analyzed using a western blot assay; B, C, the messenger RNA (mRNA) expression of p65 and Hes1 were analyzed using an real-time polymerase chain reaction (RT-PCR) assay; D, E, the proportion of β-tubulin III (TUJ-1)-positive cells was detected by flow cytometry; F, Western blot assay was carried out to evaluate the expressions of Nestin, neuron-specific enolase (NSE), TUJ-1, and microtubule-associated protein 2 (MAP2); G, enzyme-linked-immunosorbent serologic assay (ELISA) was conducted to detect the levels of cyclic adenosine monophosphate (cAMP). ** P < 0.01, *** P < 0.001 vs. control group. ### P < 0.001 vs. 3-N-butylphthalide (NBP) group.

To explore the roles of Hes1 in the neuronal differentiation process, we established cells that stably silenced Hes1 or overexpressed Hes1. The data from the Western blot assay and the RT-PCR showed that the protein and mRNA levels of p65 were not affected by either Hes1 overexpression or suppression, while Hes1 was noticeably promoted by the Hes1 plasmid and inhibited by sh-Hes1 (Figure 4A-C). Furthermore, in the Hes1-overexpression group, the percentage of TUJ-1-positive cells was lower than that in the Control group. In contrast, after transfection with sh-Hes1, the percentage of TUJ-1-positive cells was increased (Figure 4D and E). Moreover, Hes1 overexpression also inhibited nestin, NSE, TUJ-1, and MAP2 expression, while Hes1 silencing exerted the opposite effect (Figure 4F). An ELISA was conducted to assess the level of cAMP; the results revealed that the expression of cAMP was significantly reduced by Hes1 overexpression, whereas it was boosted by Hes1 silencing (Figure 4G). The results of the above experiments indicated that Hes1 had a negative effect on the neuronal differentiation of BMSCs.

### 4.5. NBP/Nuclear Factor-κB Mediates Neuronal Differentiation by Regulating Hes1

**Figure 5.**
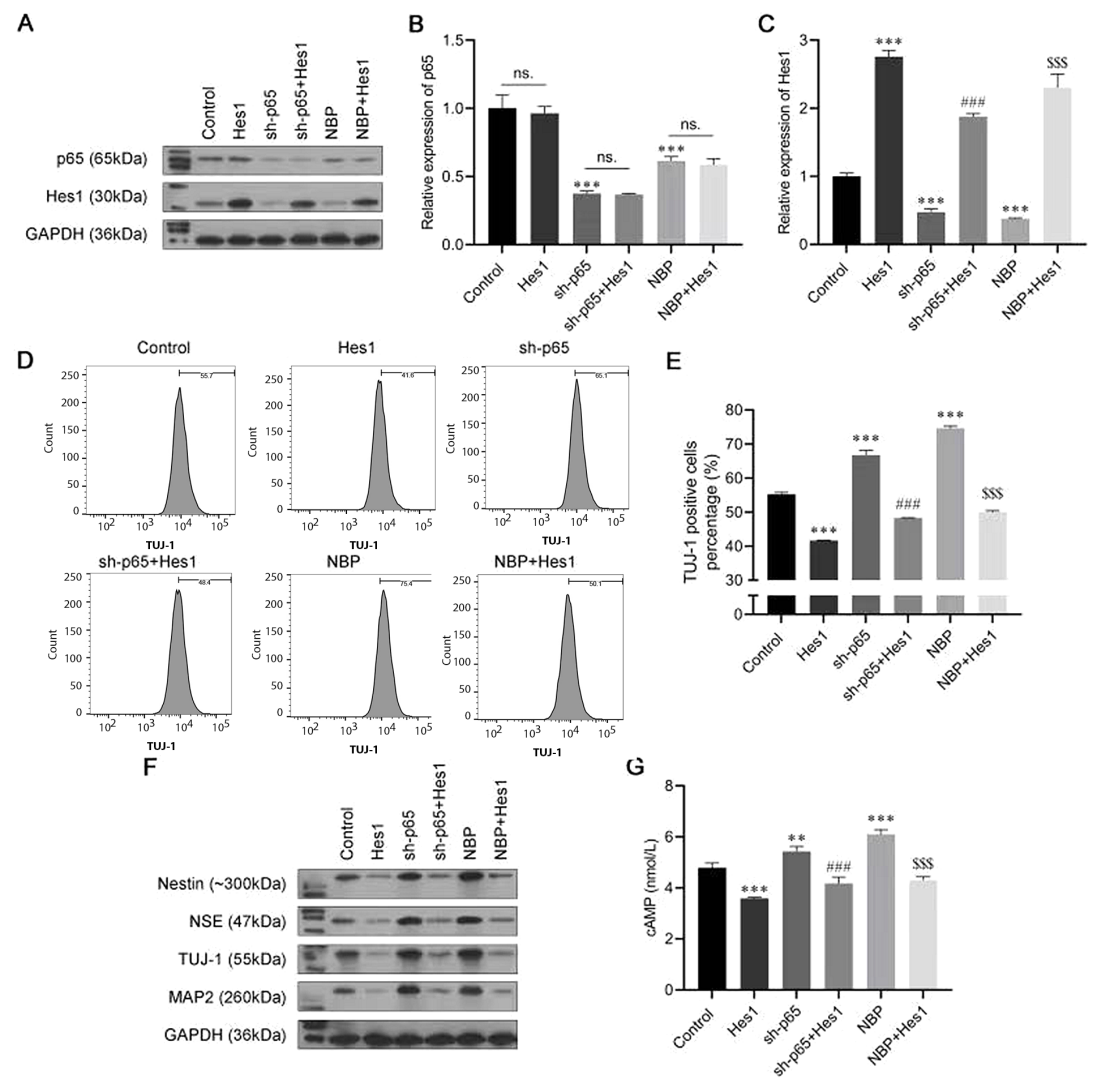
3-N-butylphthalide (NBP)/nuclear factor-κB (NF-κB) mediating neuronal differentiation by regulating Hes1. A, the protein expression of p65 and Hes1 were analyzed using a western blot assay; B, C, the messenger RNA (mRNA) expression of p65 and Hes1 were analyzed using an real-time polymerase chain reaction (RT-PCR) assay; D, E, the proportion of β-tubulin III (TUJ-1)-positive cells was detected by flow cytometry; F, Western blot assay was carried out to evaluate the expressions of Nestin, neuron-specific enolase (NSE), TUJ-1, and microtubule-associated protein 2 (MAP2); G, cyclic adenosine monophosphate (cAMP) expression was assessed by enzyme-linked-immunosorbent serologic assay (ELISA). ns, not significant. ** P < 0.01, *** P < 0.001 vs. control group. ### P < 0.001 vs. sh-p65 group. $$$ P < 0.001 vs. NBP group.

To further confirm the role of the nuclear factor-κB (NF-κB)/Hes1 axis in the progression of neuronal differentiation induced by NBP in BMSCs, a series of rescue experiments were conducted. The cells were transfected with the Hes1 overexpressing plasmid sh-p65 and the sh-p65 + Hes1 overexpressing plasmid; then, they were induced to differentiate, with or without NBP treatment. As shown in Figure 5A-C, sh-p65 and NBP had similar effects, and both suppressed the expression of Hes1. The results of the flow cytometry assay showed that the TUJ-1-positive cell rate was increased by transfection with sh-p65 or by NBP stimulation, while it was reversed by Hes1 overexpression (Figure 5D and E). Consistently, transfection with sh-p65 or NBP stimulation increased the protein levels of nestin, NSE, TUJ-1, and MAP2, but they were all recovered by Hes1 overexpression (Figure 5F). Furthermore, the promotion of cAMP by transfection with sh-p65 or NBP stimulation was also abolished by co-transfecting with the Hes1-overexpression plasmid (Figure 5G). These assays further confirmed the role of NF-κB/Hes1 in the NBP-induced neuronal differentiation of BMSCs.

## 5. Discussion

As one of the promising transplantable cells for treating neurodegenerative diseases, BMSCs can be induced to differentiate into neural cells by growth factors and cytokines. However, these strategies must be revised for current clinical applications due to limited effect and efficiency. Researchers have recently attempted to introduce cytokines or medicines to BMSCs to find more stable and effective methods to promote BMSC proliferation and differentiation. Research interest in NBP has also been generated. For instance, Lei et al. (18) found that NBP regulated the proliferation and differentiation of APP/PS1 mouse neural stem cells in vitro. In addition, NBP promoted neurogenesis and neural plasticity in rats with cerebral ischemia (19). However, this effect has not been studied in the neuronal differentiation of BMSCs.

For the first time, our study investigated the role of NBP in the differentiation of BMSCs and confirmed that NBP plays a major role in accelerating neuronal differentiation. Initially, we evaluated whether NBP affected the proliferation and apoptosis of BMSCs through a series of experiments, and the results confirmed that NBP influenced the proliferation and apoptosis of BMSCs. Nestin is the biomarker of neural precursor cells, while NSE, TUJ-1and MAP2 are the biomarkers of neurons. All are specific nerve factors and have been reported to play important roles in neural cell proliferation, apoptosis, and differentiation (20, 21). The expression levels of the above factors were analyzed to confirm neuronal differentiation. The results indicated that compared with induction treatment only, NBP-combined treatment significantly increased the protein levels of nestin, NSE, TUJ-1, and MAP2, which meant that NBP-combined treatment induced the differentiation of BMSCs to neural precursor cells and further neurons.

Neuronal apoptosis is an inevitable process in the progression of neurodegenerative diseases (22). Interestingly, we found that the expression of caspase-3, Bax, and p65 significantly decreased after NBP treatment, whereas the opposite trend was observed for Bcl-2. Additionally, the levels of IL-1β and TNF-α in the Induced + NBP group were notably lower than those of the NBP or Induced groups. Moreover, NBP also promoted cell viability in BMSCs. These results indicated that NBP had neural protection effects and might be a promising factor in inducing the neuronal differentiation of BMSCs. However, the protective effect of NBP in this study was mainly reflected in the cytotoxic resistance of b-mercaptoethanol.

As one of the subunits of NF-κB, the involvement of P65 in the apoptosis and inflammation of neural cells, as well as its contribution to neurodegenerative diseases, has been confirmed (23-25). the present study found that after single transfection with p65, the protein expression of p65 and Hes1 increased, while that of nestin, NSE, TUJ-1, and MAP2 significantly decreased. Moreover, the pro-neuronal differentiation effect of NBP was also reversed by p65 overexpression. These results indicate that the p65 signaling pathway is closely involved in the NBP-induced neuronal differentiation of BMSCs.

Evidence also indicates that Hes1 regulates neuronal differentiation (26). For instance, research shows that hypoxia prevents the premature neuronal differentiation of neural stem cells via the activation of Hes1 (27). Ochi et al. (28) indicated that the abnormal expression of Hes1 can regulate embryonic brain cell proliferation and neuronal differentiation. In the present study, the expression of nestin, NSE, TUJ-1, and MAP2 and the proportion of TUJ-1-positive cells was consistently decreased by Hes1 overexpression and increased by sh-Hes1.

The above results imply that the NF-κB/Hes1 signaling pathway is closely involved in the NBP-induced neuronal differentiation of BMSCs.

### 5.1. Conclusions

Our findings demonstrated that NBP had cytoprotective effects and promoted the neuronal differentiation of BMSCs via the NF-κB/Hes1 pathway. Therefore, our study provides a theoretical basis for further studies on the impact of NBP on the neuronal differentiation of BMSCs, which may be clinically applied in stem cell transplantation to treat neurodegenerative diseases.

However, as an in vitro experiment, the results of this study may have a limitation. The role of NBP in protecting cells and promoting neuronal differentiation of BMSCs by inhibiting the p65/Hes1 pathway needs further verification by in vivo experiments.

ijpr-22-1-132496-s001.pdf

## References

[1] Moutinho M, Codocedo JF, Puntambekar SS, Landreth GE (2019). Nuclear Receptors as Therapeutic Targets for Neurodegenerative Diseases: Lost in Translation.. Annu Rev Pharmacol Toxicol..

[2] Dewangan J, Tandon D, Srivastava S, Verma AK, Yapuri A, Rath SK (2017). Novel combination of salinomycin and resveratrol synergistically enhances the anti-proliferative and pro-apoptotic effects on human breast cancer cells.. Apoptosis..

[3] Ning K, Liu S, Yang B, Wang R, Man G, Wang DE (2022). Update on the effects of energy metabolism in bone marrow mesenchymal stem cells differentiation.. Mol Metab..

[4] Gronthos S, Mankani M, Brahim J, Robey PG, Shi S (2000). Postnatal human dental pulp stem cells (DPSCs) in vitro and in vivo.. Proc Natl Acad Sci U S A..

[5] Li Y, Liu L, Yu Z, Yu Y, Sun B, Xiao C (2021). Effects of Edaravone on Functional Recovery of a Rat Model with Spinal Cord Injury Through Induced Differentiation of Bone Marrow Mesenchymal Stem Cells into Neuron-Like Cells.. Cell Reprogram..

[6] Saxena M, Prashar P, Yadav PS, Sen J (2016). Mouse bone marrow stromal cells differentiate to neuron-like cells upon inhibition of BMP signaling.. Differentiation..

[7] Wu R, Wang N, Li M, Zang W, Xu Y (2013). Experimental study on the facilitative effects of miR-125b on the differentiation of rat bone marrow mesenchymal stem cells into neuron-like cells.. Cell Biol Int..

[8] Chen H, He Y, Chen S, Qi S, Shen J (2020). Therapeutic targets of oxidative/nitrosative stress and neuroinflammation in ischemic stroke: Applications for natural product efficacy with omics and systemic biology.. Pharmacol Res..

[9] Gao L, Guo X, Liu S, Sun Q, Qin X, Lv P (2022). DL-3-n-butylphthalide imparts neuroprotection via Nrf2/SIRT3 pathway in a mouse model of vascular dementia.. Brain Res..

[10] Li M, Meng N, Guo X, Niu X, Zhao Z, Wang W (2020). Dl-3-n-Butylphthalide Promotes Remyelination and Suppresses Inflammation by Regulating AMPK/SIRT1 and STAT3/NF-kappaB Signaling in Chronic Cerebral Hypoperfusion.. Front Aging Neurosci..

[11] Li W, Wei D, Lin J, Liang J, Xie X, Song K (2019). Dl-3-n-Butylphthalide Reduces Cognitive Impairment Induced by Chronic Cerebral Hypoperfusion Through GDNF/GFRalpha1/Ret Signaling Preventing Hippocampal Neuron Apoptosis.. Front Cell Neurosci..

[12] Lin C, Huang S, Zhang J, Yuan H, Yao T, Chen L (2022). Dl-3-N-Butylphthalide Attenuates Hypoxic Injury of Neural Stem Cells by Increasing Hypoxia-Inducible Factor-1alpha.. J Stroke Cerebrovasc Dis..

[13] Que R, Zheng J, Chang Z, Zhang W, Li H, Xie Z (2021). Dl-3-n-Butylphthalide Rescues Dopaminergic Neurons in Parkinson's Disease Models by Inhibiting the NLRP3 Inflammasome and Ameliorating Mitochondrial Impairment.. Front Immunol..

[14] Yang S, Yu C, Yang Z, Cui H, Wu Y, Liang Z (2021). DL-3-n-butylphthalide-induced neuroprotection in rat models of asphyxia-induced cardiac arrest followed by cardiopulmonary resuscitation.. J Cell Physiol..

[15] Cheng H, Huang Y, Chen W, Che J, Liu T, Na J (2021). Cyclic Strain and Electrical Co-stimulation Improve Neural Differentiation of Marrow-Derived Mesenchymal Stem Cells.. Front Cell Dev Biol..

[16] Li S, Guan H, Zhang Y, Li S, Li K, Hu S (2021). Bone marrow mesenchymal stem cells promote remyelination in spinal cord by driving oligodendrocyte progenitor cell differentiation via TNFalpha/RelB-Hes1 pathway: a rat model study of 2,5-hexanedione-induced neurotoxicity.. Stem Cell Res Ther..

[17] Fang J, Zhao X, Li S, Xing X, Wang H, Lazarovici P (2019). Protective mechanism of artemisinin on rat bone marrow-derived mesenchymal stem cells against apoptosis induced by hydrogen peroxide via activation of c-Raf-Erk1/2-p90(rsk)-CREB pathway.. Stem Cell Res Ther..

[18] Lei H, Zhang Y, Huang L, Xu S, Li J, Yang L (2018). L-3-n-Butylphthalide Regulates Proliferation, Migration, and Differentiation of Neural Stem Cell In Vitro and Promotes Neurogenesis in APP/PS1 Mouse Model by Regulating BDNF/TrkB/CREB/Akt Pathway.. Neurotox Res..

[19] Yang LC, Li J, Xu SF, Cai J, Lei H, Liu DM (2015). L-3-n-butylphthalide Promotes Neurogenesis and Neuroplasticity in Cerebral Ischemic Rats.. CNS Neurosci Ther..

[20] Barnabe GF, Schwindt TT, Calcagnotto ME, Motta FL, Martinez Jr G, de Oliveira AC (2009). Chemically-induced RAT mesenchymal stem cells adopt molecular properties of neuronal-like cells but do not have basic neuronal functional properties.. PLoS One..

[21] Gao QY, Ge J, Wang ZC, Chen HY, Huang DP, Yuan ZH (2005). [The effect of protein kinase C alpha on expression of developmental genes during differentiation of mouse embryonic stem cells into neuron-like cells in vitro].. Zhonghua Yan Ke Za Zhi..

[22] Cuenca N, Fernandez-Sanchez L, Campello L, Maneu V, De la Villa P, Lax P (2014). Cellular responses following retinal injuries and therapeutic approaches for neurodegenerative diseases.. Prog Retin Eye Res..

[23] Bellucci A, Bubacco L, Longhena F, Parrella E, Faustini G, Porrini V (2020). Nuclear Factor-kappaB Dysregulation and alpha-Synuclein Pathology: Critical Interplay in the Pathogenesis of Parkinson's Disease.. Front Aging Neurosci..

[24] Glass CK, Saijo K, Winner B, Marchetto MC, Gage FH (2010). Mechanisms underlying inflammation in neurodegeneration.. Cell..

[25] Mattson MP, Camandola S (2001). NF-kappaB in neuronal plasticity and neurodegenerative disorders.. J Clin Invest..

[26] Ohtsuka T, Ishibashi M, Gradwohl G, Nakanishi S, Guillemot F, Kageyama R (1999). Hes1 and Hes5 as notch effectors in mammalian neuronal differentiation.. EMBO J..

[27] Vecera J, Prochazkova J, Sumberova V, Panska V, Paculova H, Lanova MK (2020). Hypoxia/Hif1alpha prevents premature neuronal differentiation of neural stem cells through the activation of Hes1.. Stem Cell Res..

[28] Ochi S, Imaizumi Y, Shimojo H, Miyachi H, Kageyama R (2020). Oscillatory expression of Hes1 regulates cell proliferation and neuronal differentiation in the embryonic brain.. Development..

