# Supplementary material for: NBP Cytoprotective Effects Promoting Neuronal Differentiation in BMSCs by Inhibiting the p65/Hes1 Pathway
Source: Iran J Pharm Res. 2023 Jul 11;22(1):e132496. doi: 10.5812/ijpr-132496 (PMC10728845; doi:10.5812/ijpr-132496)
Supplement: ijpr-22-1-132496-s001.pdf [file ijpr-22-1-132496-s001.pdf]

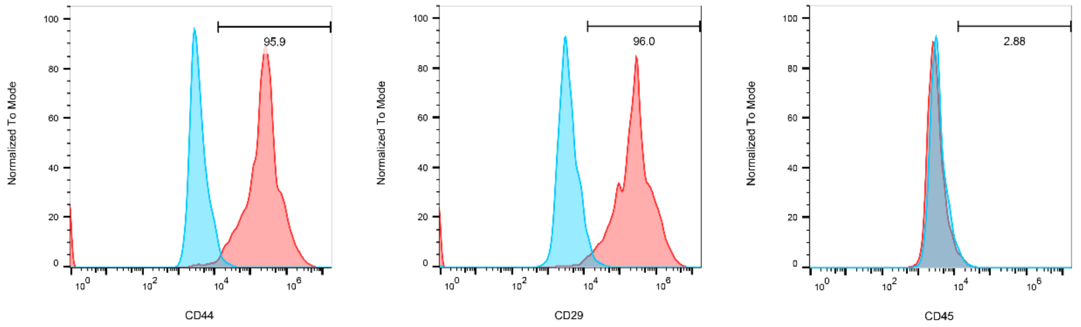

Appendix 1. Expression analysis of surface markers CD29, CD44 and CD45 in the fourth generation BMSCs.

Note: flow cytometry showed that the positive expression rate of CD29, CD44 and CD45 in the fourth generation was over 95%, and the negative expression rate of CD45.

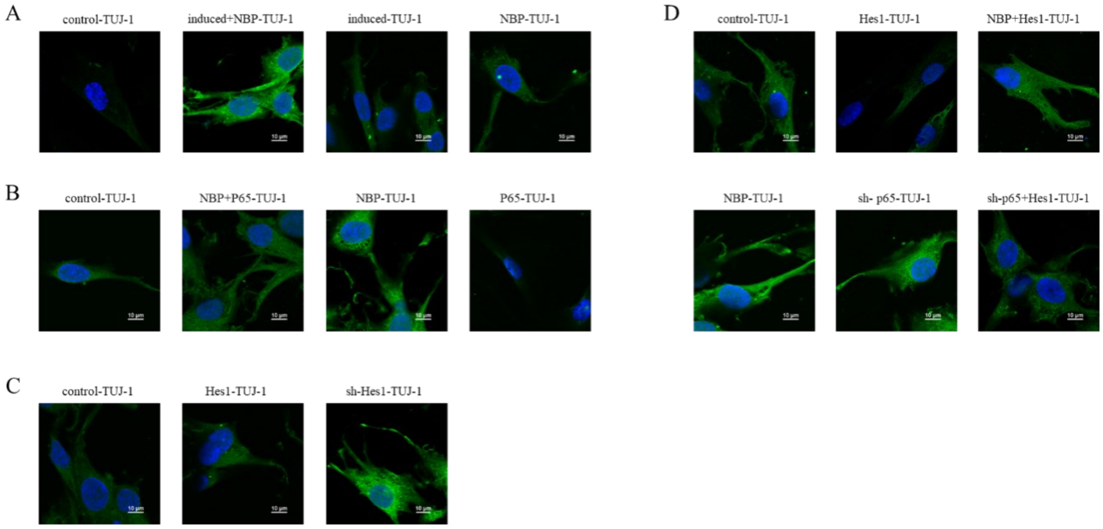

Appendix 2. Confocal microscopy of ? with DAPI counterstaining in groups. Scale bars, 10 μm.

Appendix 3. Primer Sequences of RT-qPCR Assay

**Gene**	**Sequence (5'- 3')**
**P65 F**	ATGTGGAGATCATTGAGCAGC
**P65 R**	CCTGGTCCTGTGTAGCCATT
**Hes1 F**	TCAATGCCATGACCTACCCC
**Hes1 R**	AAACACCTTAGCCGCCTCTC
**GAPDH F**	TGTTCGTCATGGGTGTGAAC
**GAPDH R**	ATGGCATGGACTGTGGTCAT
